# Psychological complications of childhood chronic physical illness in Nigerian children and their mothers: the implication for developing pediatric liaison services

**DOI:** 10.1186/1753-2000-2-34

**Published:** 2008-11-19

**Authors:** Muideen O Bakare, Olayinka O Omigbodun, Olugbenga B Kuteyi, Martin M Meremikwu, Ahamefule O Agomoh

**Affiliations:** 1 Child and Adolescent Unit, Federal Neuro-Psychiatric Hospital, New Haven, Enugu, Enugu State, Nigeria; 2 Department of Psychiatry, College of Medicine, University of Ibadan, Nigeria; 3 Child and Adolescent Unit, Federal Psychiatric Hospital, Calabar, Nigeria; 4 Department of Pediatrics, College of Medical Sciences, University of Calabar, Nigeria; 5 General/Forensic Psychiatry Unit, Federal Neuro-Psychiatric Hospital, New Haven, Enugu, Enugu State, Nigeria

## Abstract

**Background:**

Pediatric liaison services attending to the psychological health needs of children with chronic physical illness are limited or virtually non-existent in Nigeria and most sub-Saharan African countries, and psychological problems complicate chronic physical illness in these children and their mothers. There exist needs to bring into focus the public health importance of developing liaison services to meet the psychological health needs of children who suffer from chronic physical illness in this environment. Sickle cell disease (SCD) and juvenile diabetes mellitus (JDM) are among the most common chronic physical health conditions in Nigerian children. This study compared the prevalence and pattern of emotional disorders and suicidal behavior among Nigerian children with SCD, JDM and a group of healthy children. Psychological distress in the mothers of these children that suffer chronic physical illness was also compared with psychological distress in mothers of healthy control children.

**Methods:**

Forty-five children aged 9 to 17 years were selected for each group of SCD, JDM and controls. The SCD and JDM groups were selected by consecutive clinic attendance and the healthy children who met the inclusion criteria were selected from neighboring schools. The Youth version of the Computerized Diagnostic Interview Schedule for Children, version IV (C- DISC- IV) was used to assess for diagnosis of emotional disorders in these children. Twelve-item General Health Questionnaire (GHQ – 12) was used to assess for psychological distress in mothers of these children and healthy control children.

**Results:**

Children with JDM were significantly more likely to experience DSM – IV emotional disorders than children with SCD and the healthy group (p = 0.005), while children with JDM and SCD were more likely to have 'intermediate diagnoses' of emotional disorders (p = 0.0024). Children with SCD and JDM had higher rates of suicidal ideation when compared to healthy control children and a higher prevalence of maternal psychological distress was found in their mothers when compared to the mothers of healthy children (p = 0.035).

**Conclusion:**

The higher prevalence of emotional disorders and suicidal ideation among children with SCD and JDM points to a need for development of liaison services in pediatric facilities caring for children with chronic physical illness to ensure holistic approach to their care. The proposed liaison services would also be able to provide family support interventions that would address the psychological distress experienced by the mothers of these children.

## Background

Pediatric liaison services attending to the psychological health needs of children with chronic physical illness are virtually non existent in most parts of Nigeria and other sub-Saharan African countries. Childhood chronic physical illness is one of the major concerns in pediatric population in this environment and childhood chronic physical conditions are complicated by psychological problems, which do not only affect the children but also impact on the psychological health of the mothers who bear mostly the burden of care. Assessing the magnitude of the problem would help bring into focus the public health importance of designing policies and liaison services to meet the psychological health needs of children who suffer from chronic physical illness in this environment. Such liaison services could also address the psychological health needs of mothers of these children through family support interventions. Sickle cell disease (SCD) and juvenile diabetes mellitus (JDM) are among the most common chronic childhood physical illness in Nigeria.

### Sickle cell disease

SCD is found prevalent among people of African descent and Arabs. It is known to put an enormous psychosocial burden on both the patients and caregivers [1-3]. It is a hereditary and chronic medical condition that includes homozygous sickle cell disease (Hb.SS), sickle cell hemoglobin C disease (Hb.SC) and sickle cell B thalassaemia (SB.Thal.) [4]. SCD is characterized by anemia, chronic organ damage, acute episodes of vaso-occlusive crises, infection, splenic sequestration, and acute chest syndrome among others [4]. Homozygous sickle cell disease (Hb. SS) is the most common type of SCD and has the most debilitating prognosis [4]. In Nigeria, sickle cell disease (SCD) occurs in about two percent of the pediatric population [5].

Most studies on psychological disorders among sickle cell patients have focused on adult and young adult patients with SCD [6-8]. One of the few studies assessing for psychiatric morbidity among Nigerian children and adolescents with SCD was carried out in a hospital setting in the south east region of Nigeria. Using Rutter's Behavior Questionnaires [9], the rate of psychiatric morbidity as determined by parents and teachers' reports were about twenty seven and twenty three percent respectively. However, these rates were not based on current diagnostic criteria of International Classification of Diseases, Tenth Edition (ICD-10) [10] or Diagnostic and Statistical Manual of Mental Disorders, Fourth Edition (DSM-IV) [11], and there are suggestions that parents or teachers may not be able to accurately report internalizing symptoms such as anxiety and depression in the children [12-14]. This study attempts to overcome these limitations by using a structured instrument that generates diagnosis based on DSM-IV criteria to determine the rates of specific anxiety and depressive disorders by interviewing the children themselves. In addition, rather than looking at all children with SCD, only children with homozygous sickle cell disease (Hb.SS), which is the most common and severe form of SCD in this environment were studied.

### Juvenile diabetes mellitus

Another important chronic physical illness in childhood in this environment is juvenile diabetes mellitus (JDM). In Nigeria, the cumulative prevalence rate (CPR) of insulin dependent diabetes mellitus (IDDM) had been reported to range from 0.038% and 0.025% for boys and girls respectively between the ages of 5 – 17 years [15]. Several studies carried out in the developed world report psychological problems such as depression and anxiety in patients with JDM [16-21]. There is a dearth of information regarding specific psychological disorders associated with JDM in children and adolescents in Nigeria.

### Maternal mental health

A closely related determinant of the outcome of chronic medical conditions in children and adolescents is maternal mental health. Maternal mental health is an important factor for family cohesion and this had been found to influence treatment compliance in child and adolescent patients with JDM in particular [19,22].

For these two childhood-chronic physical conditions and others that start in childhood, it is important to develop pediatric liaison services that would enable putting in place interventions to improve the short and long term outcome of childhood chronic physical illness.

This study determined and compared the prevalence and pattern of emotional disorders among children and adolescents with SCD and JDM attending specialist clinics in two hospitals located in the south-south region of Nigeria. Suicidal behavior among these children and adolescents and maternal mental distress were also assessed.

## Methods

### Location and participants

The sample consisted of 135 children and adolescents aged 9 to 17 years, with diagnoses of SCD (N = 45) and JDM (N = 45) and healthy children (N = 45) who served as controls. Consecutive SCD and JDM patients who had been diagnosed for one year or more, aged between 9 and 17 years and attending the outpatient clinic of the University of Calabar Teaching Hospital, Nigeria and the General Hospital, Calabar, Nigeria were included in the study.

In addition to the clinical history obtained from the records of these children, each patient with SCD had hemoglobin electrophoresis done to confirm homozygous sickle cell disease (Hb.SS). JDM patients had been diagnosed by the attending physician and must have been attending the clinic on follow-up visits for a minimum of one year. The diagnoses made were based on Fasting Blood Glucose (FBG) of greater than 7.8 mmol/L (140 mg/dl) recorded on two occasions.

In the study group, children who did not have homozygous sickle cell disease (Hb.SS), children who required emergency treatment and children whom their mothers or both parents were not primarily responsible for their care were excluded from the study.

The healthy children were selected from a nearby public primary and secondary schools. A total of 402 children from the two schools met our age range inclusion criterion. Recruitment was made by asking the pupils that were willing to participate in the study to leave their names with their teachers. A total of 105 pupils out of which 82 pupils met our other inclusion criteria, indicated their interest. Out of these 82 pupils, 45 were randomly selected by balloting putting into consideration the need to match for sex. Subjects who had sibling(s) with any chronic medical condition based on clinical history among the healthy group were excluded from the study. Pupils whom their mothers or both parents were not primarily responsible for their care were excluded from the study because of the need to interview the mothers. The minimum educational level of mothers of children involved in the study was elementary school completed, those mothers who did not have any formal education were excluded from the study.

### Ethical consideration

Permission for this study was obtained from the ethical committee of the University of Calabar Teaching Hospital, (UCTH), Calabar, Nigeria. Patients, healthy children and their parents were duly informed about the intention of the study and availability of help or treatment for any diagnosed psychological problem. Consent was obtained from the patients, healthy controls and their parents before the interviews were conducted.

## Materials

### National Institute of Mental Health (NIMH) Computerized – Diagnostic Interview Schedule for Children, Version 4 (C-DISC-IV) [23]

The Computerized-Diagnostic Interview Schedule for Children, version 4 (C-DISC-IV) was used. The C-DISC-IV is a highly structured clinical interview schedule that generates diagnoses based on DSM-IV criteria. The Youth version of C-DISC-IV, Clinician Assisted module was used for the interview of the study clients.

The major depressive disorder, dysthymic disorder, generalized anxiety disorder, separation anxiety disorder and social phobia modules of C-DISC-IV were used to conduct interviews among children selected for the study. In addition, Suicidal Ideation (Past Year), Suicidal Plan (Past Year) and Life-time Suicide Attempt were generated from the clinical diagnostic report for each participant, produced from the C-DISC-IV computer algorithm program.

The C-DISC-IV was administered to the study groups and controls in the original English version. There was no difficulty experienced in doing this, possibly because the children involved in the study were students mostly in secondary schools in Nigerian environment where medium of instruction in schools is English language.

A positive diagnosis was assigned if the child/adolescent met the full symptom (duration and frequency) criteria as specified in DSM-IV while an 'intermediate diagnosis' was assigned when at least half of the symptom criteria specified in DSM-IV were met.

### General Health Questionnaire, (GHQ) [24]

This is a self-administered screening instrument that is used in detection of non-specific psychiatric disorders. The GHQ-12 was administered to the mothers of children studied to detect evidence of psychological distress. The twelve-item version of GHQ was chosen because it had been validated for use in this environment and is short and easy to complete. The standard GHQ method of scoring 0-0-1-1 for each item was employed, which allows a maximum score of 12. In a validity study of the GHQ-12 in this environment, a cut-off of 2 was obtained as the optimum threshold with sensitivity of 77.8% and specificity of 79.4% [25]. The cut-off point of 2 and above was chosen for this study. GHQ-12 had been used across cultures to assess non-specific psychiatric disorders [26]. Validity of GHQ-12 in assessing for psychiatric disorders against various standardized interview schedules that make diagnosis according to ICD – 10 [10] and DSM – IV [11] criteria had been documented [26,27].

The minimum educational level of mothers of children involved in this study was elementary school completed and they were able to complete the English version of GHQ-12 questionnaires without assistance aside the adequate explanation on how to complete the questionnaires.

### Data analysis

The data were analyzed using the SPSS (Statistical Package for Social Sciences), Version 15.0. Qualitative data were analyzed using Chi-square test and quantitative inter-group data were analyzed using one-way analysis of variance (ANOVA). Significant alpha value (p) was ≤ 0.05.

## Results

### Demographic information of the children

A total of 70 males (51.8%) and 65 females (48.2%) were involved in the study. There were forty-five (45) children in each group of SCD, JDM and healthy controls.

There were no significant differences in the gender distribution among the study participants and healthy children (χ^2 ^= 0.95, df = 2, p = 0.621). The age range of the study participants was from 9 to 17 years. The mean age for sickle cell disease patients was 13.76 ± 2.74 years, for juvenile diabetic patients it was 14.96 ± 1.94 years, and for the healthy group it was

14.11 ± 2.74 years. The mean age for the total sample was 14.27 ± 2.54 years. There was no statistical significant difference in the mean age of the three groups of children using one way analysis of variance (F-Ratio = 2.73, p = 0.07).

### Demographic information of the mothers

#### Age distributions

The mean ages of the mothers of children involved in the study were 40.56 ± 3.60 years, 40.36 ± 3.26 years and 39.20 ± 2.69 years for mothers of children with SCD, JDM and healthy controls respectively. There was no significant difference in the mean age of the mothers in the three groups using one way analysis of variance (F- Ratio = 2.35, p = 0.10).

#### Educational level

The minimum educational level of mothers involved in the study was elementary school education completed. Twenty (44.4%) of mothers of children with SCD had college education, while 25 (55.6%) did not. Twenty four (53.3%) of mothers of children with JDM had college education, while 21 (46.7%) did not. Eighteen (40.0%) of mothers of healthy children had college education, while 27 (60.0%) did not. There was no significant difference in the mothers' level of education (χ^2 ^= 1.67, df = 2, p = 0.434).

#### Marital status

Forty one (91.1%) of mothers of children with SCD were married, while 4 (8.9%) were single parents. Thirty-three (73.3%) of mothers of children with JDM were married, while 12 (26.7%) were single parents. For mothers of healthy children, 38 (84.4%) were married and 7 (15.6%) were single parents either due to being separated or divorced from their spouse. There was no statistical significant difference in the marital status distribution in the three groups (χ^2 ^= 5.14, df = 2, p = 0.077). Table 1 showed the demographic information of the children and their mothers.

**Table 1:** Demographic information of the children and their mothers

**Demographic Information**	**SCD** **N = 45**	**JDM** **N = 45**	**HEALTHY GROUP** **N = 45**
**Children's Gender**			
• Male	26 (57.8%)	22 (48.9%)	22 (48.9%)
• Female	19 (42.2%)	23 (51.1%)	23 (51.1%)
			
**Children's Mean Age (Years)**	13.76 ± 2.74	14.96 ± 1.94	14.11 ± 2.74
			
**Mothers' Mean Age (Years) **	40.56 ± 3.60	40.36 ± 3.26	39.20 ± 2.69
			
**Mothers' Educational Level**			
• Below College Education	25 (55.6%)	21 (46.7%)	27 (60.0%)
• College Education	20 (44.4%)	24 (53.3%)	18 (40.0%)
			
**Mothers' Marital Status**			
• Married	41 (91.1%)	33 (73.3%)	38 (84.4%)
• Single Parent	4 (8.9%)	12 (26.7%)	7 (15.6%)

SCD: Homozygous Sickle Cell Disease Patients

JDM: Juvenile Diabetic Patients

#### Prevalence and pattern of DSM-IV emotional disorders

When the five specific emotional disorders assessed for were pooled together, 2 (4.4%) of the SCD patients and 9 (20.0%) of the JDM patients met the criteria for one or more DSM-IV diagnoses of emotional disorder. One (2.2%) of children among the healthy group met the criteria for one DSM-IV diagnosis. Three of the JDM patients had co-morbid diagnoses of social phobia and major depressive disorder. Children with JDM were significantly more likely to have DSM-IV emotional disorders than children with SCD and the healthy group (χ^2 ^= 10.3, df = 2, p = 0.005). The prevalence and pattern of DSM-IV emotional disorders is shown in Table 2.

**Table 2:** Prevalence and pattern of DSM – IV emotional disorders

**DSM-IV Emotional Disorders**	**SCD** **N = 45** **N (%)**	**JDM** **N = 45** **N (%)**	**HEALTHY GROUP** **N = 45** **N (%)**
**SAD**	-	-	-

**SP**	-	8 (17.8)	-

**MDD**	1 (2.2)	3 (6.7)	1 (2.2)

**DD**	1 (2.2)	1 (2.2)	-

**GAD**	-	-	-

**(SAD + SP + MDD + DD + GAD) The Five Emotional Disorders**	**2 (4.4)**	**9 (20.0)**	**1 (2.2)**

SCD: Homozygous Sickle Cell Disease Patients

JDM: Juvenile Diabetic Patients

SAD: Separation Anxiety Disorder

SP: Social Phobia

DD: Dysthymic Disorder

MDD: Major Depressive Disorder

GAD: Generalized Anxiety Disorder

#### Prevalence and pattern of 'intermediate diagnoses' of emotional disorders

When the five specific emotional disorders assessed for were pooled together, 17 (37.8%) of SCD patients, 19 (42.2%) of JDM patients and 5 (11.1%) of the healthy subjects had 'intermediate diagnoses' of one or more of the five emotional disorders.

Nine of the SCD patients had co-morbid 'intermediate diagnoses' of social phobia and major depressive disorder, while one had co-morbid 'intermediate diagnoses' of social phobia and dysthymic disorder. Five of the JDM patients had co-morbid 'intermediate diagnoses' of social phobia and major depressive disorder, while three had co-morbid 'intermediate diagnoses' of social phobia and dysthymic disorder. One of the healthy children had co-morbid 'intermediate diagnoses' of social phobia and major depressive disorder, while another of the healthy children had co-morbid 'intermediate diagnoses' of social phobia and dysthymic disorder.

Children with JDM and SCD were more likely to have higher rate of 'intermediate diagnoses' of the five emotional disorders assessed for (χ^2 ^= 12.05, df = 2, p = 0.0024).

The prevalence and pattern of 'intermediate diagnoses' of the five emotional disorders among JDM, SCD and healthy control children are shown in Table 3.

**Table 3:** Prevalence and pattern of 'intermediate diagnoses' of emotional disorders

**'Intermediate Diagnoses' of Emotional Disorders**	**SCD** **N = 45** **N (%)**	**JDM** **N = 45** **N (%)**	**HEALTHY GROUP** **N = 45** **N (%)**
**SAD**	5 (11.1)	6 (13.3)	1 (2.2)

**SP**	10 (22.2)	9 (20.0)	3 (6.7)

**MDD**	9 (20.0)	5 (11.1)	1 (2.2)

**DD**	1 (2.2)	5 (11.1)	1 (2.2)

**GAD**	2 (4.4)	2 (4.4)	1 (2.2)

**(SAD + SP + MDD + DD + GAD) The Five Emotional Disorders**	**17 (37.8)**	**19 (42.2)**	**5 (11.1)**

SCD: Homozygous Sickle Cell Disease Patients

JDM: Juvenile Diabetic Patients

SAD: Separation Anxiety Disorder

SP: Social Phobia

DD: Dysthymic Disorder

MDD: Major Depressive Disorder

GAD: Generalized Anxiety Disorder

### Suicidal behavior

#### Suicidal ideation (past year), suicidal plan (past year) and lifetime suicide attempt

Nine (20.0%) SCD patients and 5 (11.1%) JDM patients had suicidal ideation in the past one year while none of the healthy subjects expressed such idea. There was a significant difference in the prevalence of suicidal ideation among the three groups with patients with SCD and JDM showing more suicidal ideation than the healthy group (χ^2 ^= 13.52, df = 2, p = 0.001). One (2.2%) SCD patient and 1 (2.2%) JDM patient had a definite plan in the past one year to commit suicide and had also made a life-time suicide attempt. No healthy subject had a definite plan to commit suicide in the past one year or ever made a lifetime suicide attempt.

#### Psychological distress in the mothers

Twenty eight (62.2%) mothers of SCD children, 24 (53.3%) mothers of JDM children and 16 (35.6%) mothers of the healthy control children had GHQ-12 score of 2 and above. This difference was statistically significant with mothers of SCD and JDM children more likely to experience psychological distress compared to mothers of the healthy control children (χ^2 ^= 6.72, df = 2, p = 0.035).

## Discussion

This cross sectional study among Nigerian children with homozygous sickle cell disease (SCD), juvenile diabetes mellitus (JDM), healthy children and their mothers is a testimony to the fact that psychological problems complicate childhood chronic physical illness and confirms that emotional disorders are more prevalent among children with chronic medical illness.

Looking at specific DSM-IV anxiety and depressive disorders, and their relationship to these two childhood chronic illnesses certain similarities and differences were found. For the three anxiety disorders studied, no subject in any of the three groups met the DSM-IV diagnostic criteria for these disorders except for social phobia found in about eighteen percent of children with JDM. Children with DSM-IV depressive disorders were few among children with JDM, SCD and the healthy group.

However with a less stringent diagnostic criteria in form of an 'intermediate diagnosis', greater numbers of children with SCD and JDM had social phobia and separation anxiety disorder. A fifth of children with SCD and over a tenth with JDM had 'intermediate diagnoses' of major depressive disorder and then over a tenth of children with JDM had 'intermediate diagnoses' of dysthymic disorder. Looking at the five specific emotional disorders together, about forty two percent of children with JDM and thirty eight percent of children with SCD had 'intermediate diagnoses' of one or more emotional disorders and this was significantly more than what was found among the healthy group of children.

With regard to suicidal behavior, children with JDM and SCD were more likely to have suicidal ideation than healthy control children.

Psychological distress was significantly higher and more prevalent among mothers of children with childhood chronic physical illness than mothers of healthy control children.

Prevalence rates of psychiatric morbidity found in studies among children and adolescents with SCD, in this environment and other parts of the world ranges between twenty three and twenty nine percent [9,28]. With the Children's Depression Inventory, Yang et al [28] obtained a prevalence of depression among children with SCD of twenty nine percent. This rate was almost half (fifteen percent) for the same set of children when clinical interviews were used, suggesting that the less stringent the diagnostic criteria the higher the prevalence rates. The rate obtained by use of the depression inventory is closer to the rate of depression obtained in this study when the 'intermediate diagnostic' criteria were employed.

Using the 'intermediate diagnostic' criteria, the prevalence rate for emotional disorders assessed in this study for children with SCD is even higher (about thirty eight percent) than what was obtained in an earlier study carried out in south east Nigeria [9] in which the Rutter Behavior Questionnaires were used to assess for psychiatric morbidity using parents' (twenty six percent) and teachers' reports (about twenty three percent). This may further substantiate the observation that children and adolescents are better reporters of internalizing symptoms they are experiencing [12-14].

The prevalence rate of twenty and approximately forty two percent for emotional symptoms found among JDM children in this study using DSM-IV and 'intermediate diagnoses' criteria respectively are comparable to psychiatric morbidity rates ranging between thirty three and forty eight percent found among children and adolescents with JDM in other parts of the world [17,21].

Prevalence of emotional disorders was higher in most instances among children with JDM than those with SCD. However, there were twice as many SCD children with suicidal ideation as JDM children and in regard to maternal mental distress there were no significant differences between the two groups of disorders.

With the higher rates of emotional disorders among children with JDM compared to SCD, it may be tempting to speculate that children with JDM suffer more distress from their physical illness compared to their SCD counterparts. This may be explicable by the fact that children with SCD are known to have intervals of healthy periods when they do not have crises and all they have to do is take regular oral medications which include prophylactic anti-malarial and hematinics [4]. This appears easier and less distressing to cope with when compared to children with JDM who may need to inject themselves with doses of insulin two to three times daily as the case may be [29].

That children with SCD had experienced in the past year, twice as much suicidal ideation than children with JDM could be explained by the recurrent bone pain crises which could be very excruciating and often characterized presentation of symptoms in children with SCD. Could it be that during these periods of experiencing excruciating pains, the affected children with SCD wish they were dead? A close association had also been found between pain and suicidal behavior [30,31]. Pain management in sickle cell crises in Nigeria may need a review of practice and policy because analgesics like morphine and other highly potent opium that could aid immediate pain alleviation are not commonly available when prescribed and when available, affordability in terms of cost is often the problem because the healthcare financing system is still largely out of pocket payment.

The higher prevalence of psychological distress found among the mothers of children with SCD and JDM when compared to mothers of healthy children can be related to previous studies that reported association between maternal mental health and behavioral problems in the children [32-34]. Maternal mental health as a factor of family cohesion had been reported to influence treatment compliance in child and adolescent patients with diabetes mellitus [19,22]. The need to develop pediatric liaison services that can see to family support interventions for families of children with childhood chronic physical illness can not be under played. Family support interventions had been shown to be beneficial to the mental health of mothers of children with childhood chronic illness [35].

## Limitations

It is not abnormal in some sub-Saharan African subcultures including Nigeria that parents could put their children under the guardian care of close relatives like aunties, uncles or grand parents who may become primarily responsible for the care and well being of such children because of possible economic reason among others. The exclusion of children whom were not living with either their mothers or both patents and whom their mothers or both parents were not primarily responsible for their care could have some influence on the prevalence of emotional disorders found among the children in this study and this may limit the generalization of the findings. It is however more likely that, those children that were not living with their parents would experience more psychological problems than those living primarily with their parents. The non-inclusion of mothers that were illiterate could also be a limitation in generalizing the findings of this study. Being educated is often an indicator of better socio-economic status in this environment and it is more likely that the group of mothers and children who do not have formal education that were excluded from the study would experience more psychological problems possibly because of confounding factor of low socio-economic status. Omigbodun [36] in an earlier study in south west Nigeria had found that psychosocial issues like separation from the primary parents to live with relatives, economic problems among other factors contributed to developing several child psychiatric disorders. Another limitation was that severity of the primary medical problems was not assessed in the children with chronic physical illness and this could have enhanced the findings of the study. Though not statistically significant, the differences in the mean ages of the children and their mothers and maternal marital status between groups which were approaching statistical significance could have had some influence on the findings of the study.

However, these are not envisaged as limitations that would significantly impact on the implication of the findings of the study which is the need for developing viable pediatric liaison services in an environment where child and adolescent mental health care is given little or no attention.

## Conclusion

The limited or virtually non-existent of pediatric liaison services that address the psychological health needs of children with chronic physical illness in this environment throw question at the readiness of our mental health policies in the area of addressing psychosocial needs of children with chronic physical illness. The findings of this study and other previous studies in this environment [3,9,37] that had documented psychosocial adjustment problems in children with chronic physical illness are pointing at the need to develop pediatric liaison services across Nigeria that would address the psychosocial issues in children and adolescents with chronic physical illness and possibly care for the psychological health needs of mothers of these children who mostly bear the burden of care. This would help the process of adjustment in the children and their mothers and would contribute to improving overall prognosis.

## Competing interests

The authors declare that they have no competing interests.

## Authors' contributions

All authors contributed to the conception of the study. MOB, OOO, OBK and MMM were involved in writing and revision of the manuscript. All authors approved the final draft of the manuscript.
